# Plasma calcitonin gene-related peptide levels in idiopathic intracranial hypertension: an exploratory study

**DOI:** 10.1186/s10194-024-01799-y

**Published:** 2024-06-04

**Authors:** Nik Krajnc, Florian Frank, Stefan Macher, Martin Michl, Nina Müller, Sarah Maier, Sina Zaic, Christian Wöber, Berthold Pemp, Gregor Broessner, Gabriel Bsteh

**Affiliations:** 1 Department of Neurology, Medical University of Vienna, Vienna, Austria; 2 Comprehensive Center for Clinical Neurosciences and Mental Health, Medical University of Vienna, Vienna, Austria; 3 Department of Neurology, Headache Outpatient Clinic, Medical University of Innsbruck, Anichstrasse 35, Innsbruck, 6020 Austria; 4 Department of Ophthalmology, Medical University of Vienna, Vienna, Austria; 5 Institute of Medical Statistics and Informatics, Medical University of Innsbruck, Innsbruck, Austria

**Keywords:** Idiopathic intracranial hypertension, Calcitonin gene-related peptide, Headache, Migraine, CGRP

## Abstract

**Background:**

Idiopathic intracranial hypertension (IIH) is a debilitating condition characterized by increased intracranial pressure often presenting with chronic migraine-like headache. Calcitonin gene-related peptide (CGRP) plays an important pathophysiological role in primary headaches such as migraine, whilst its role in IIH has not yet been established.

**Methods:**

This longitudinal exploratory study included patients with IIH, episodic migraine (EM) in a headache-free interval and healthy controls (HC). Blood samples were collected from a cubital vein and plasma CGRP (pCGRP) levels were measured by standardized ELISA.

**Results:**

A total of 26 patients with IIH (mean age 33.2 years [SD 9.2], 88.5% female, median BMI 34.8 kg/m^2^ [IQR 30.0–41.4]), 30 patients with EM (mean age 27.6 years [7.5], 66.7% female) and 57 HC (mean age 25.3 years [5.2], 56.1% female) were included. pCGRP levels displayed a wide variation in IIH as well as in EM and HC on a group-level. Within IIH, those with migraine-like headache had significantly higher pCGRP levels than those with non-migraine-like headache (F_(2,524)_ = 84.79; *p* < 0.001) and headache absence (F_(2,524)_ = 84.79; *p* < 0.001) throughout the observation period, explaining 14.7% of the variance in pCGRP levels. CGRP measurements showed strong intraindividual agreement in IIH (ICC 0.993, 95% CI 0.987–0.996, *p* < 0.001). No association was found between pCGRP levels and ophthalmological parameters.

**Conclusions:**

Although interindividual heterogeneity of pCGRP levels is generally high, migraine-like headache seems to be associated with higher pCGRP levels. CGRP may play a role in the headache pathophysiology at least in a subgroup of IIH.

## Introduction

Idiopathic intracranial hypertension (IIH) is a debilitating condition characterized by increased intracranial pressure, most commonly occurring in young obese women, causing chronic headaches and papilledema with a risk of permanent vision loss [1 [1]]. Its incidence is increasing with rising obesity rates worldwide [2], as even modest weight gain is associated with an increased risk of developing IIH [3]. While IIH can be a devastating condition leading to visual impairment or even blindness, these risks can often be avoided if diagnosis and treatment are made at an early stage of the disease and papilledema is carefully monitored. However, IIH is also associated with reduced quality of life due to severe headaches that interfere with patients’ daily activities. More than half of patients with IIH experience persistent headache even after resolution of the papilledema and normalization of intracranial pressure [4–6]. Why this is the case remains poorly understood.

Calcitonin gene-related peptide (CGRP) is a 37 amino acid regulatory neuropeptide and potent vasodilator [2], comprising two forms in humans, $\alpha$-CGRP and $\beta$-CGRP [3], both of which share structural similarity and 94% homology as well as identical binding affinity and intensity [4]. $\alpha$-CGRP is found in the central and peripheral nervous system, and is primarily produced and stored in A$\delta$- and C-fiber sensory neurons in the trigeminal ganglion and dorsal root ganglia [4, 5], playing a pivotal role in the pathophysiology of migraine [6]. Upon activation of nociceptors, CGRP is released into the innervated tissues, where it contributes to neurogenic inflammation, leading to arterial vasodilation [4]. It is then taken up by postcapillary vessels, appearing in the circulation [7], where it is considered to be a biomarker of various pain disorders, including primary headache disorders such as migraine [8]. In migraine patients, CGRP plasma levels are elevated during migraine attacks, and migraine-like attacks can be provoked by infusion of CGRP [9].

Monoclonal antibodies targeting CGRP or its receptor have not only dramatically changed the landscape of migraine, but also seem to be effective in reducing headache in IIH [10].

Given these circumstances, an exploratory study was undertaken to determine the role of CGRP in IIH.

## Methods

### Patients and definitions

For this exploratory study, we prospectively included patients with newly diagnosed IIH according to Friedman criteria from an ongoing prospectively followed observational cohort study (Vienna Idiopathic Intracranial Hypertension Biomarker study [VIIH-BIO]), jointly conducted by the Department of Neurology and Department of Ophthalmology at the Medical University of Vienna starting in January 2021.

Data are collected at the following time points: at diagnosis (D0) and after one day (D1), one (W1) and two weeks (W2), and one (M1), two (M2), three (M3) and six months (M6). Afterwards, patients are followed in a three-month interval. Briefly, standardized VIIH-BIO case reports include demographic data, disease specific parameters as well as documentation of diagnostic and therapeutic procedures. Specialized neurologists and neuro-ophthalmologists perform all examinations. Headache diagnosis is assessed by a headache specialist also using a specific headache diary. Headache phenotype is classified according to ICHD-3 [11]. All patients are treated according to best practice based on recommendation of weight loss, pharmacological treatment with acetazolamide, topiramate and/or furosemide, and invasive treatment options such as serial lumbar punctures and subsequent ventriculoperitoneal (VP) shunt or optic nerve sheath fenestration (ONSF) in case of visual-threatening treatment-refractory papilledema [12].

For the present study, the following data were obtained at each time point: body weight, monthly headache days (MHD) with headache phenotype recorded in a specific headache diary (including changes to those phenotypes during follow-up), and ophthalmological assessment including visual acuity, perimetry, fundoscopy, optical coherence tomography (OCT) and ocular ultrasonography. Patients with IIH with migraine history did not have a migraine attack for at least six months prior to the inclusion in the study or had < 1 migraine headache day on average in the last three months.

Patients with episodic migraine (EM) in a headache-free interval [17] and healthy controls (HC) from the Department of Neurology, Medical University of Innsbruck, Austria, served as control groups in the cross-sectional part of the study. In the EM group, only patients with ≥ 3 consecutive migraine- and headache-free days prior to blood sampling were included. Diagnosis of any primary headache syndrome except for infrequent episodic tension-type headache served as an exclusion criterion for HC. Importantly, none of the patients were on active preventatives or any CGRP targeting therapies.

### Ophthalmological assessment

Best-corrected visual acuity was assessed using Sloan charts at distance after subjective refraction. Results were given in logarithm of the minimum angle of resolution (logMAR). Meaningful change was defined as ≥ 0.2 logMAR [13].

For perimetry, we performed automated visual field testing (Humphrey Field Analyzer, Carl Zeiss Meditec, Jena, Germany) using 30 − 2 Swedish Interactive Threshold Algorithm (SITA) standard protocols, quantifying the mean deviation in decibels (dB) of all test locations compared to age-matched controls and defining abnormal perimetry as a mean deviation lower than − 2 dB.

Fundoscopy included assessment of absence or presence of papilledema and secondary optic atrophy. We used the Frisén staging scale to rate papilledema severity, categorizing the swelling of optic discs from stage 0 (no papilledema) to stage 5 (severe papilledema) [14].

For OCT imaging, we used the same spectral-domain OCT (Spectralis OCT, Heidelberg Engineering, Heidelberg, Germany; software Heidelberg eye explorer software version 6.9a) adhering to the OSCAR-IB quality control criteria and describing findings in accordance with the APOSTEL criteria [15, 16]. For peripapillary retinal nerve fiber layer (pRNFL) measurement, a 12° (3.4 mm) ring scan centered on the optic nerve head was used (1536 A-scans, automatic real-time tracking [ART]: 100 averaged frames) [17]. Ganglion cell layer (GCL) volume was measured without pupil dilatation in both eyes of each patient by means of a 20°×20° macular volume scan (centered on the macula with 512 A-scans and 25 B-scans aligned vertically with 16 averaged frames). Volume values characterize the mean volume of the circular area centered around the foveola, corresponding to the 6 mm ring of the circular grid defined by the Early Treatment Diabetic Retinopathy Study [18]. Image processing was semiautomated using the built-in proprietary software for automated layer segmentation and manual correction of obvious errors. Measurements of worse eyes were used for statistical analysis, i.e., higher pRNFL thickness as a marker of oedema and lower GCL volume as a marker of neuroaxonal loss.

For assessment of the optic nerve sheath diameter (ONSD), we performed transbulbar sonography (ABSolu, Quantel Medical, Cournon d’Auvergne, France) after topical anesthesia with oxybuprocaine eye drops. We performed quantitative measurement of ONSD using standardized amplitude modulation (A-scan) echography with tissue sensitivity settings, placing the 8 MHz A-scan probe on the temporal eye equator in primary gaze position [19, 20]. At least two measurements were taken within 3 mm of the posterior bulb wall, and the highest was documented as the diameter.

### Blood collection and separation of plasma

Blood was taken from a cubital vein using EDTA-K tubes (S-Monovetten, Sarstedt, Nümbrecht, Germany) and centrifuged at 4 °C for 5 min with 3,000 rpm/5,000 g. The plasma fraction was taken off with an Eppendorf pipette, transferred to cryovials (Nunc CryoTubes, Merck, Darmstadt, Germany), frozen in liquid nitrogen and stored at 80 °C within 10–12 min after blood sampling.

### CGRP sample processing

For detailed information on the rigid (pre-)processing and analysis of the samples, please refer to our previous article [21]. In short, samples were processed with a double-antibody sandwich enzyme-linked immunosorbent assay (ELISA; CGRP Enzyme Immunoassay #A05481, shortly named CGRP EIA, Bertin Bioreagent, Montigny-le-Bretonneux, France) for α- and β-CGRP, with a cross-reactivity with amylin, calcitonin and substance P of < 0.01%. For this purpose, a synthetic interstitial solution was prepared, and protease inhibitors were added to create a standard buffer, which was fitted with human CGRP and diluted to create serial dilutions of CGRP. Furthermore, human blood plasma was used as an alternative to the standard buffer. With these preparations a reference curve was fitted to later determine the individual CGRP concentrations of each sample. Detailed information on the processing and analysis of the sample is available elsewhere [21].

### Standard protocol approvals, registrations, patient consents, and reporting

The study was approved by the ethics committee of the Medical University Vienna (ethical approval number: 2216/2020). Written informed consent was obtained from all patients. This study adheres to the reporting guidelines outlined within the Strengthening the Reporting of Observational Studies in Epidemiology (STROBE) Statement.

### Data availability statement

Data supporting the findings of this study are available from the corresponding author upon reasonable request by a qualified researcher and upon approval by the data-clearing committee of the Medical University Vienna.

### Statistics

Statistical analysis was performed using SPSS 26.0 (SPSS Inc, Chicago, IL, USA). Categorical variables were expressed in absolute frequencies and percentages, continuous parametric variables as mean and standard deviation (SD) and continuous non-parametric variables as median with inter-quartile range (IQR) or absolute range (AR) as appropriate. Continuous variables were tested for normal distribution using Kolmogorov–Smirnov test with Lilliefors correction. Logarithmic transformation was used to reduce the skewness of the data. Univariate comparisons were performed by the chi-square test, one-way analysis of variance (ANOVA), and Kruskal-Wallis test with pairwise comparisons as appropriate. Univariate correlation analyses were performed using the Pearson or Spearman test as appropriate. Repeated measures ANOVA was used to determine the change in pCGRP levels over time, adjusted for age, sex, body mass index [BMI], headache duration and migraine history.

To test the intraindividual variability of CGRP levels, the intraclass correlation coefficient (ICC) was calculated based on a mean-rating, absolute-agreement, 2-way mixed-effects model.

Prespecified sensitivity analyses to determine the potential confounding influence were performed with the same statistical analysis setup excluding patients with IIH without papilledema (IIH-WOP).

Missing values were handled by multiple (20 times) imputation using the missing not at random (MNAR) approach with pooling of estimates according to Rubin’s rules [22].

The significance level was set at a two-sided *p*-value < 0.05.

## Results

**Fig. 1:**
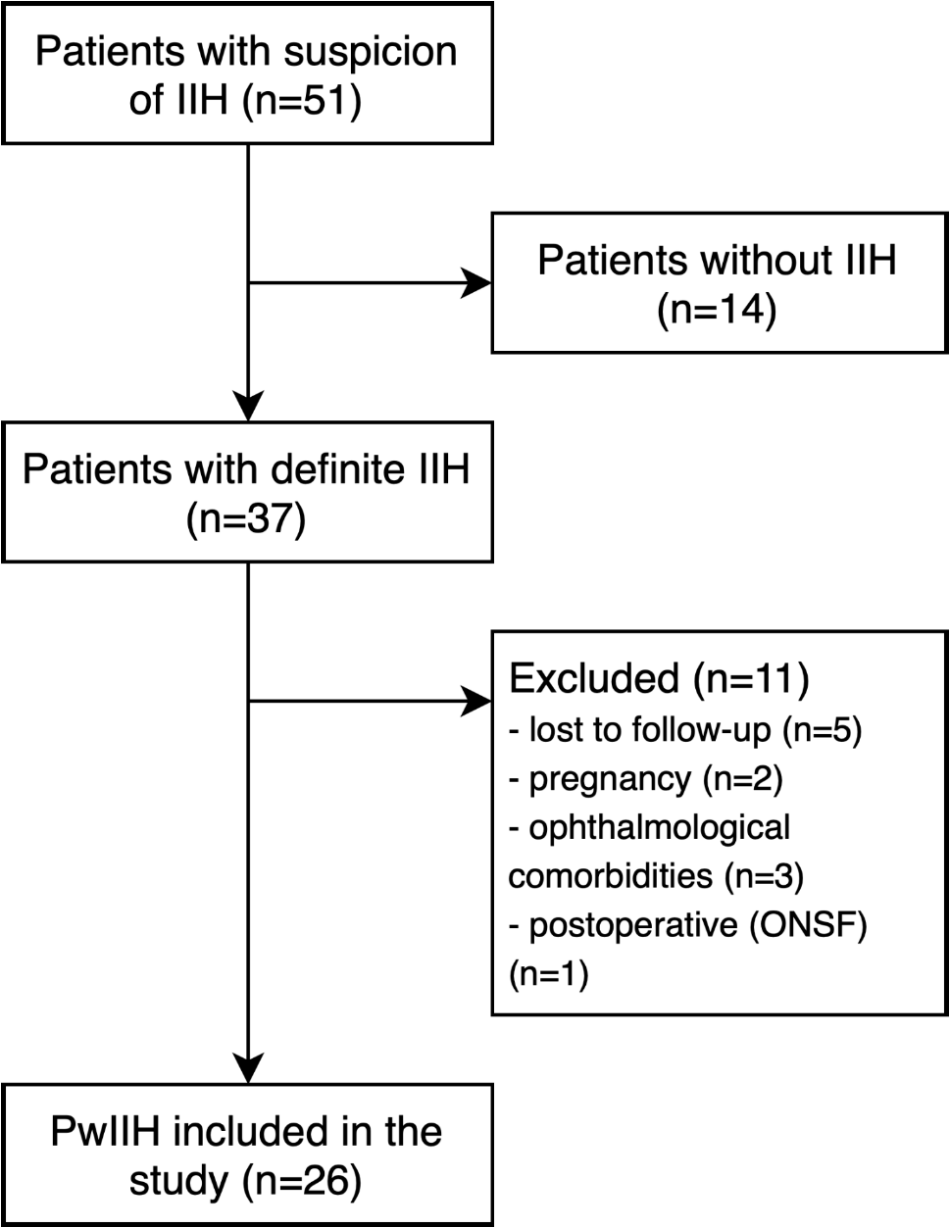
Flow chart of inclusion/exclusion process. IIH: idiopathic intracranial hypertension, ONSF: optic nerve sheath fenestration

A total of 26 patients with IIH were enrolled in the VIIH-BIO study as of November 23rd, 2022 (mean age of 33.2 years [SD 9.2], 88.5% female, median BMI 34.8 kg/m^2^ [30.0–41.4]). The inclusion/exclusion process is depicted in Fig. 1. As controls, 30 patients with EM in a headache-free interval (mean age 27.6 years [7.5], 66.7% female), and 57 HC (mean age 25.3 years [5.2], 56.1% female) were recruited. Patients with EM and HC were significantly younger and more often male than patients with IIH (both *p* < 0.001). Detailed clinical characteristics of the IIH study cohort at baseline are given in Table 1.

**Table 1:** Clinical characteristics of the IIH study cohort at baseline

	Patients with IIH (*n* = 26)
Female^a^	23 (88.5)
Age at diagnosis (years)^b^	33.2 (9.2)
IIH-WOP^a^	6 (23.1)
BMI^c^	34.8 (30.0, 41.4)
CSF opening pressure (cmH_2_O)^d^	30 (22, 41)
Visual disturbances^a^	18 (69.2)
Pulsatile tinnitus^a^	11 (42.3)
**Headache**	
Headache presence^a^	19 (73.1)
Migraine-like headache^a^	13 (50.0)
Monthly headache days^d^	12 (0, 28)
Chronic headache^a^	11 (42.3)
Migraine history^a^	8 (30.8)
**Ophthalmological outcomes**	
Frisén-Scale^d^	1 (0, 4)
Visual acuity of the worse eye^d^	1.20 (0.06, 1.25)
Decreased visual acuity^a^	1 (3.8)
Visual field mean deviation of the worse eye (dB)^c^	-1.38 (-3.29, -0.38)
Abnormal visual field^a^	9 (34.6)
pRNFL thickness of the worse eye (µm)^c^	118.5 (100.5, 188.5)
GCL volume of the worse eye (mm^3^)^c^	1.07 (1.01, 1.15)
ONSD of the worse eye (mm)^b^	5.63 (0.75)

BMI: body mass index, CSF: cerebrospinal fluid, GCL: ganglion cell layer, IIH: idiopathic intracranial hypertension, IIH-WOP: idiopathic intracranial hypertension without papilledema, ONSD: optic nerve sheath diameter, pRNFL: peripapillary retinal nerve fiber layer

^a^Number (percentage), ^b^Mean (standard deviation), ^c^Median (interquartile range), ^d^Median (range)

### Cross-sectional data

**Fig. 2:**
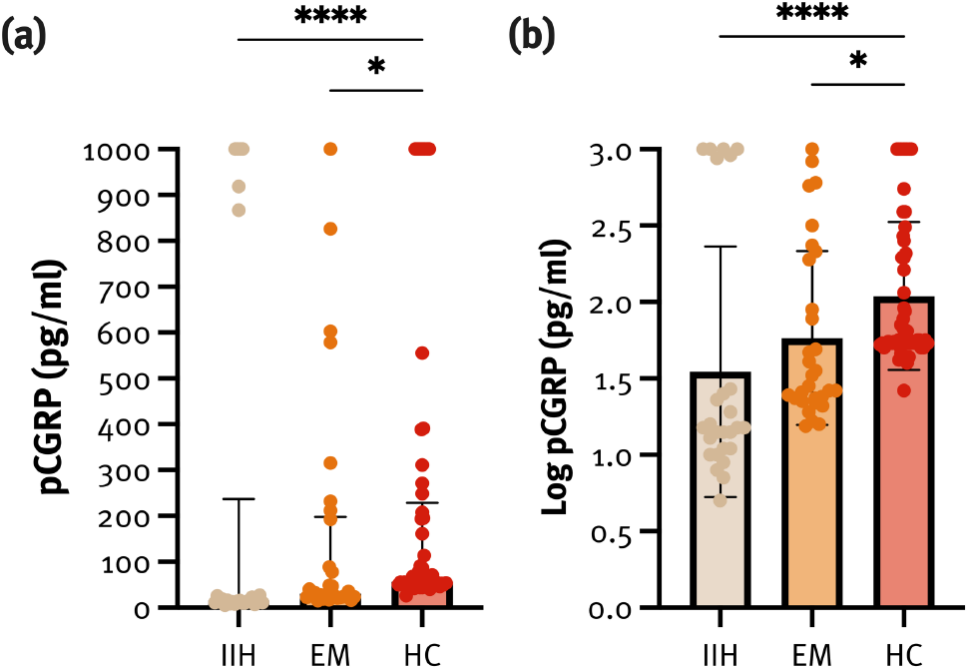
Baseline plasma CGRP (pCGRP) levels (pg/ml) (**a**) and log pCGRP levels (**b**) in patients with idiopathic intracranial hypertension (IIH), episodic migraine (EM) in a headache-free interval, and healthy controls (HC).

pCGRP levels showed wide variation within all three groups: IIH (median 15 pg/ml, 95% CI 10.8–237), EM (30.5 pg/ml, 95% CI 23.2–197.4), and HC (56.3 pg/ml, 95% CI 53.4–228.2) (Fig. 2). Notably, patients with IIH with a history of migraine had significantly higher median pCGRP levels than those without migraine (1,000 pg/ml [25–1,000] vs. 14 pg/ml [11–17], *p* < 0.001). CSF opening pressure did not correlate with pCGRP levels at baseline (r_s_= − 0.17, *p* = 0.423).

#### Longitudinal data in IIH

**Fig. 3:**
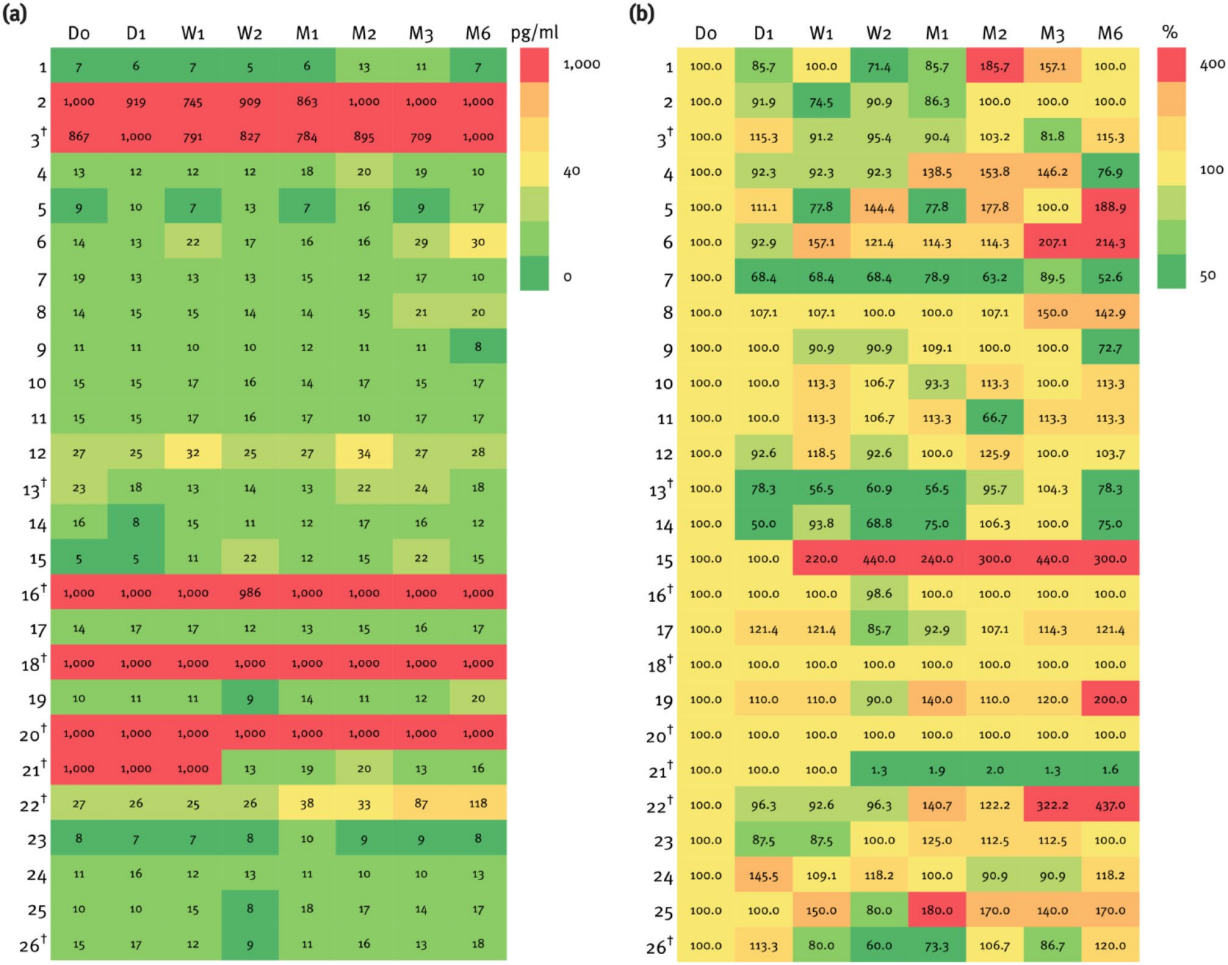
Heat map illustrating the longitudinal distribution of pCGRP levels (pg/ml) among patients with IIH (1–26). pCGRP levels are displayed as colors ranging from green to red as shown in the legend; absolute values of pCGRP levels (**a**) and relative change compared to baseline (**b**). The x-axis shows different time points with D0 as baseline (diagnosis), followed by day one (D1), week one (W1) and two (W2), and month one (M1), two (M2), three (M3) and six (M6). Missing data were imputed for 21/208 (10.1%) time points

In patients with IIH, no significant change in mean CGRP levels was observed during the observation period (F_(1, 528)_ = 3.73; *p* = 0.231). Furthermore, the ICC for CGRP measurements showed excellent intraindividual agreement in patients with IIH (ICC 0.993, 95% CI 0.987–0.996, *p* < 0.001) and migraine (ICC 0.999, 95% CI 0.998–1.000, *p* < 0.001); no longitudinal data were available for HC. Individual CGRP levels for each patient are shown in Fig. 3.

### ^†^Patients with migraine history

#### Headache outcomes in IIH

**Fig. 4:**
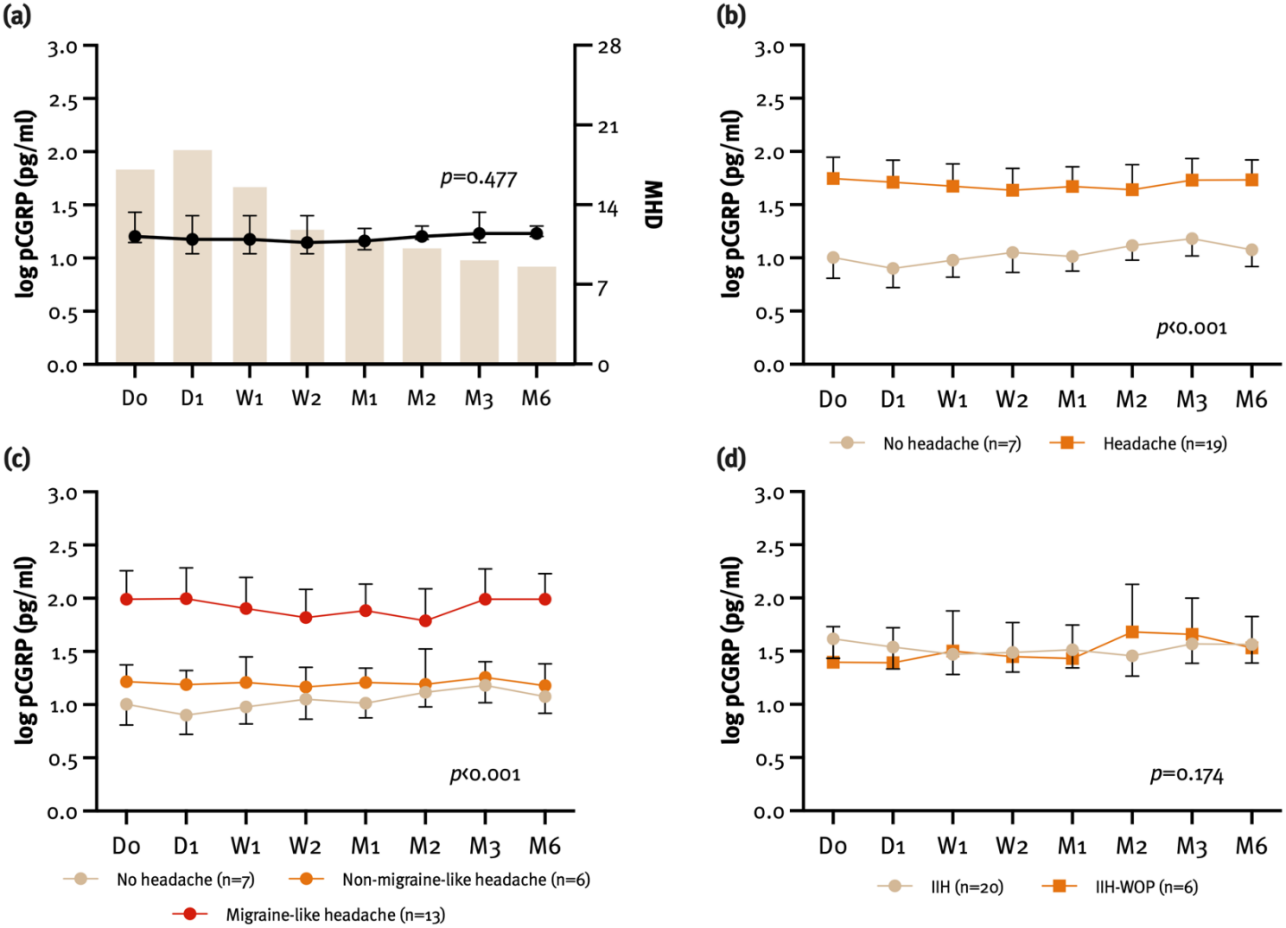
Longitudinal association between pCGRP levels (pg/ml) and monthly headache days (MHD) (**a**), headache presence (**b**), headache phenotype (**c**) and IIH without papilledema (IIH-WOP) (**d**)

Patients with IIH with headache (*n* = 19) had significantly higher pCGRP levels compared to those without headache (*n* = 7; F_(1, 528)_ = 105.38; *p* < 0.001). This association remained significant in the multivariate model after adjustment for migraine history (F_(1, 527)_ = 25.60; *p* < 0.001), with headache explaining 4.6% of the variance in pCGRP levels. Furthermore, migraine-like headache was independently associated with higher pCGRP levels compared to both non-migraine-like headache and headache absence (F_(2, 524)_ = 84.79; *p* < 0.001), explaining 14.7% of the variance in pCGRP levels in the multivariate model. However, pCGRP levels were not associated with headache frequency as per MHD (β= -0.06; 95% CI -0.02, 0.01; *p* = 0.477) (Fig. 4). pCGRP levels und log pCGRP levels in patients with IIH according to the headache phenotype are shown in Table 2. None of the patients changed their headache phenotype during the observation period. Prespecified sensitivity analyses excluding patients with IIH-WOP did not significantly alter the overall results or impact individual variables.

### Ophthalmological outcomes

Papilledema degree was not associated with pCGRP levels (β= -0.03; 95% CI -0.16, 0.13; *p* = 0.798), neither did pCGRP levels significantly differ between patients with IIH and IIH-WOP (F_(1, 528)_ = 1.86; *p* = 0.174) (Fig. 4). Also, neither visual acuity (β = 0.08; 95% CI -0.63, 1.72; *p* = 0.359) nor visual field (β = 0.15; 95% CI -0.01, 0.09; *p* = 0.142) of the worse eye were associated with pCGRP levels. In OCT, neither pRNFL thickness (β = 0.07; 95% CI -0.01, 0.01; *p* = 0.496) nor GCL volume (β = 0.04; 95% CI -1.05, 1.58; *p* = 0.690) were associated with pCGRP levels. Finally, pCGRP levels were also not associated with ONSD in transbulbar sonography (β= -0.05; 95% CI -0.21, 0.10; *p* = 0.487).

Prespecified sensitivity analyses excluding patients with IIH-WOP did not significantly alter the overall results or impact individual variables.

**Table 2:** Plasma CGRP (pCGRP) levels (**a**) and log pCGRP levels (**b**) in patients with idiopathic intracranial hypertension (IIH) according to the headache phenotype

	pCGRP (pg/ml)^a^				log pCGRP (pg/ml)^b^			
	Migraine-like headache (*n* = 13)	Non-migraine-like headache (*n* = 6)	No headache (*n* = 7)	*p*-value	Migraine-like headache (*n* = 13)	Non-migraine-like headache (*n* = 6)	No headache (*n* = 7)	*p*-value
D0	25 (14–1,000)	15 (13–23)	9 (7–16)	**< 0.001**	1.99 (0.93)	1.22 (0.14)	1.00 (0.20)	**< 0.001**
D1	19 (15–1,000)	14 (12–24)	8 (6–13)	**< 0.001**	1.86 (0.89)	1.20 (0.14)	0.95 (0.17)	**< 0.001**
W1	22 (17–850.5)	15 (12–17)	11 (7–13)	**< 0.001**	1.88 (0.84)	1.19 (0.16)	0.99 (0.13)	**< 0.001**
W2	17 (13–863)	16 (10–20.2)	13 (8–17)	**< 0.001**	1.85 (0.90)	1.16 (0.20)	1.06 (0.20)	**< 0.001**
M1	118 (14–832.5)	17 (12–27)	15 (10–16)	**< 0.001**	1.98 (0.86)	1.21 (0.26)	1.10 (0.15)	**< 0.001**
M2	87.9 (15–834.3)	17 (11–23.1)	12 (10.3–13.9)	**< 0.001**	1.97 (0.86)	1.23 (0.20)	1.07 (0.11)	**< 0.001**
M3	31.0 (20–884.7)	15.1 (9.5–27.8)	13.9 (8–17)	**< 0.001**	1.97 (0.91)	1.22 (0.27)	1.04 (0.35)	**< 0.001**
M6	29.5 (13.5–1,000)	16 (11–19)	8.5 (7–10)	**< 0.001**	1.98 (0.77)	1.20 (0.21)	0.92 (0.08)	**< 0.001**

^a^Median (IQR), ^b^Mean (SD)

## Discussion

Aiming to investigate the role of pCGRP levels in IIH, we observed a large interindividual variation in IIH comparable to that in EM and HC cross-sectionally, but a high intraindividual stability of CGRP levels longitudinally. Notably, patients with IIH with migraine history and/or displaying migraine-like headache had significantly higher pCGRP levels throughout the observation period.

The development of CGRP as a biomarker and treatment target in migraine has revolutionized the landscape of headache research [23, 24]. Migraine and IIH appear to be closely related: both share important risk factors such as female gender and obesity, the headache in IIH often resembles the migraine phenotype, and both respond to treatment with topiramate [25, 26]. Because of these similarities, it has been hypothesized that CGRP may also play a role in the pathophysiology of IIH-related headache [27]. CGRP is released upon stimulation of primary meningeal afferents, leading to vasodilation of intracranial arteries responsible for pain perception, as evidenced by elevated CGRP levels during and between migraine attacks [28, 29]. In support of this hypothesis, a series of seven patients with IIH with migraine-like headache who were successfully treated with anti-CGRP monoclonal antibodies has been recently published [30], implicating the CGRP pathway as a mechanistic driver of headache in IIH. A possible pathophysiological mechanism may be a nociceptive trigeminal firing at congested dural venous sinuses, leading to activation of the trigeminovascular system and subsequent pain sensitization [31, 32]. Trigeminovascular terminals appear to be widespread throughout the dural venous sinuses, particularly at the openings of the bridge veins, and contain large amounts of CGRP and other neuropeptides [33]. Distension of the intracranial vessels leads to the release of CGRP which, by binding to its receptors on neurons and glial cells, triggers neurogenic inflammation, a process similar to that seen during a migraine attack [34].

Our findings contribute to the increasing body of evidence linking CGRP and headache in IIH. We found evidence that patients with migraine-like headache features in IIH have higher pCGRP levels regardless of their migraine history. Although median pCGRP levels were significantly lower at baseline in the whole IIH cohort compared to controls, we observed a wide interindividual variation in IIH comparable to that in HC and EM, but a high intraindividual stability of CGRP levels longitudinally. Patients with IIH with migraine history and/or displaying migraine phenotype headache had significantly higher CGRP levels throughout the observation period.

The latter were significantly younger and more often male, which may have likely influenced our results due to the known association between age, sex and pCGRP levels [35]. Furthermore, pCGRP levels may be negatively influenced by BMI in the same way as serum neurofilament light chains (sNfL) [36], which may have also contributed to lower pCGRP levels in some patients with IIH. Thus, it seems unlikely that patients with IIH generally have lower pCGRP levels than patients with EM and/or HC, but rather this could be due to confounding factors not taken into account in the cross-sectional data analysis. On the contrary, we found that there are some patients with IIH that displayed high pCGRP levels. Despite the lack of association between pCGRP levels and MHD, this is consistent with recent studies [35, 37]. Additionally, it may be speculated that CGRP is produced, released and/or degraded at different rates in different individuals possibly explaining the huge variance on an individual level.

Most interestingly, CGRP levels were not associated with the degree of papilledema or other parameters of the degree of intracranial pressure in our cohort. This strengthens the hypothesis that pathophysiological pathways involving CGRP may partially explain the well-known puzzle of poor correlation between intracranial pressure and headache in IIH [27, 38, 39].

As we have recently shown, migraine-like headache is an independent predictor of adverse headache outcome in IIH even after resolution of papilledema, i.e., when intracranial pressure is significantly reduced if not normalized [39]. This may be explained by higher CGRP levels observed in a recent study [40]. Our findings support these results and additionally show that pCGRP levels are not associated with ophthalmological outcomes and only seem elevated in patients with migraine-like headache, confirming a potential common pathophysiological pathway in a subgroup of IIH and migraine.

We hypothesize that a subset of individuals with IIH may have an increased sensitivity or susceptibility to CGRP pathways, leading to a migraine-like pain experience and possibly clinical response to CGRP targeting therapies. Measurement of pCGRP may provide a practical and clinically feasible approach to identify this subgroup and provide insight into both prognosis and treatment strategy.

Several limitations of this study need to be acknowledged: the exploratory study design with a rather small sample size (due to the rarity of IIH) prevented more complex statistical models, and some missing values during the follow-up due to the observational study design, which were mitigated by the imputation of missing values. Due to the limited dataset, we were unable to fully match patients with EM and HC. Importantly, it is not possible to objectively distinguish between IIH and migraine as independent comorbidities as opposed to IIH with a migraine-like headache phenotype. Thus, it could be that patients with IIH with elevated pCGRP levels in our cohort have migraine as an independent coexisting comorbidity, explaining the elevated pCGRP levels in IIH. However, even after adjusting the model for covariates including migraine history, headache remained associated with higher pCGRP levels. Also, no data about headache intensity or headache presence during the blood sampling were systematically collected. As some authors argue that patients with IIH-WOP should be excluded or investigated separately when studying IIH, we performed prespecified sensitivity analyses excluding patients with IIH-WOP, which did not significantly change the results giving strength to our data. Finally, there was a ceiling effect of extreme pCGRP levels as samples were not further diluted, which seemed sufficient for the exploratory nature of the study.

## Conclusion

This study is a pioneering investigation into the development of pCGRP levels in IIH and sheds light on its potential role in the pathophysiology of headache in this disorder. First, interindividual heterogeneity of pCGRP levels is high in IIH as well as in EM and HC, while intraindividually, pCGRP levels seem to be quite stable. Second, migraine-like headache seems to be associated with higher pCGRP levels. Thus, CGRP may play a role in the headache pathophysiology at least in a subgroup of IIH. Third, it is noteworthy that pCGRP levels do not seem to be associated with ophthalmological parameters, which again challenges the conventional explanation linking intracranial pressure and headache in IIH.

Further studies should focus on measuring pCGRP levels in IIH, which may be a promising and clinically feasible way to identify relevant subgroups and inform both prognosis and treatment strategy.

## Data Availability

Data supporting the findings of this study are available from the corresponding author upon reasonable request by a qualified researcher and upon approval by the data-clearing committee of the Medical University Vienna.

## Abbreviations

ANOVA: Analysis of Variance
AR: Absolute Range
ART: Automatic Real-Time Tracking
BMI: Body Mass Index
CGRP: Calcitonin Gene-Rlated Peptide
CSF: Cerebrospinal Fluid
dB: Decibels
ELISA: Enzyme-Linked Imunosorbent Assa
EM: Episodic Migraine
GCL: Ganglion Cell Layer
HC: Healthy Controls
ICC: Intraclass Correlation Coefficient
ICHD: International Classification of Headache Disorders
IIH: Idiopathic Intracranial Hypertension
IIH-WOP: Idiopathic Intracranial Hypertension Without Papilledema
IQR: Interquartile Range
logMAR: Logarithm of the Minimum Angle of Resolution
MHD: Monthly Headache Days
MNAR: Missing Not At Random
OCT: Optical Coherence Tomography
ONSD: Optic Nerve Sheath Diameter
ONSF: Optic Nerve Sheath Fenestration
pCGRP: Plasma Calcitonin Gene-Related Peptide
pRNFL: Peripapillary Retinal Nerve Fiber Layer
SD: Standard Deviation
SITA: Swedish Interactive Threshold Algorithm
STROBE: Strengthening the Reporting of Observational Studies in Epidemiology
VIIH-BIO: Vienna Idiopathic Intracranial Hypertension Biomarker
VP: Ventriculoperitoneal
